# A novel subtilase with NaCl-activated and oxidant-stable activity from *Virgibacillus *sp. SK37

**DOI:** 10.1186/1472-6750-11-65

**Published:** 2011-06-09

**Authors:** Ekkarat Phrommao, Jirawat Yongsawatdigul, Sureelak Rodtong, Montarop Yamabhai

**Affiliations:** 1 School of Food Technology, Institute of Agricultural Technology, Suranaree University of Technology, 111 University Avenue, Nakhon Ratchasima, 30000, Thailand; 2 School of Microbiology, Institute of Science, Suranaree University of Technology, 111 University Avenue, Nakhon Ratchasima, 30000, Thailand; 3 School of Biotechnology, Institute of Agricultural Technology, Suranaree University of Technology, 111 University Avenue, Nakhon Ratchasima, 30000, Thailand

## Abstract

**Background:**

Microbial proteases are one of the most commercially valuable enzymes, of which the largest market share has been taken by subtilases or alkaline proteases of the *Bacillus *species. Despite a large amount of information on microbial proteases, a search for novel proteases with unique properties is still of interest for both basic and applied aspects of this highly complex class of enzymes. Oxidant stable proteases (OSPs) have been shown to have a wide application in the detergent and bleaching industries and recently have become one of the most attractive enzymes in various biotechnological applications.

**Results:**

A gene encoding a novel member of the subtilase superfamily was isolated from *Virgibacillus *sp. SK37, a protease-producing bacterium isolated from Thai fish sauce fermentation. The gene was cloned by an activity-based screening of a genomic DNA expression library on Luria-Bertani (LB) agar plates containing 1 mM IPTG and 3% skim milk. Of the 100,000 clones screened, all six isolated positive clones comprised one overlapping open reading frame of 45% identity to the *aprX *gene from *Bacillus *species. This gene, designated *aprX-sk37 *was cloned into pET21d(+) and over-expressed in *E. coli *BL21(DE3). The enzyme product, designated AprX-SK37, was purified by an immobilized metal ion affinity chromatography to apparent homogeneity and characterized. The AprX-SK37 enzyme showed optimal catalytic conditions at pH 9.5 and 55°C, based on the azocasein assay containing 5 mM CaCl_2_. Maximum catalytic activity was found at 1 M NaCl with residual activity of 30% at 3 M NaCl. Thermal stability of the enzyme was also enhanced by 1 M NaCl. The enzyme was absolutely calcium-dependent, with optimal concentration of CaCl_2 _at 15 mM. Inhibitory effects by phenylmethanesulfonyl fluoride and ethylenediaminetetraacetic acid indicated that this enzyme is a metal-dependent serine protease. The enzyme activity was sensitive towards reducing agents, urea, and SDS, but relatively stable up to 5% of H_2_O_2_. Phylogenetic analysis suggested that AprX-SK37 belongs to a novel family of the subtilase superfamily. We propose the name of this new family as alkaline serine protease-X (AprX).

**Conclusions:**

The stability towards H_2_O_2 _and moderately halo- and thermo-tolerant properties of the AprX-SK37 enzyme are attractive for various biotechnological applications.

## Background

Microbial proteases are one of the most commercially valuable enzymes [1], of which the largest market share has been taken by subtilases or alkaline proteases of the *Bacillus *species, which has been extensively studied in terms of both biological properties and applications [1-3]. Subtilase is the superfamily of subtilisin-like serine proteases [4] in the clan SB of serine peptidases, family S8, according to the MEROPS database [5]. It is one of the biggest clans of serine peptidases and ubiquitously distributed in various organisms, including bacteria, archaea, eukaryotes, and viruses [6]. Based on amino acid sequence similarity, they are classified into six families: subtilisins, thermitase, proteinase K, lantibiotic peptidase, kexin, and pyrolysin [4]. Subtilisins are further classified into six subfamilies namely, true subtilisins, high-alkaline proteases, intracellular proteases [4], phylogenetically intermediate subtilisins (PISs) [7,8], high molecular mass subtilisins (HMSs) [9,10], and oxidant stable proteases (OSPs) [11,12]. The latter subfamily, OSPs, has been shown to have a wide application in the detergent and bleaching industries and has recently become one of the most attractive enzymes in various biotechnological applications. [8,11-17].

Despite the tremendous amount of information on microbial proteases, a search for novel proteases with unique properties is still of interest for both basic and applied aspects of these highly complex class of enzymes [2]. In addition to a classical method of screening for a new microorganism harbouring an interesting protease from various environments [2], different molecular biology techniques, such as directed evolution [18,19], site directed mutagenesis [20] and metagenomic analysis [21], have been used to engineer numerous proteases with improved or novel properties. By applying DNA technology, it is essential that the gene of the protease of interest is cloned, engineered and expressed in an efficient host.

In this research, a genomic library of *Virgibacillus *sp. SK37, a moderately halophilic bacterium isolated from Thai fish sauce [22] was constructed and screened for heterologous protease expression in *Escherichia coli*. After an extensive screening, one open reading frame encoding a gene similar to *aprX *was obtained. This gene was cloned, over-expressed in *E. coli *and purified by immobilized metal ion affinity chromatography (IMAC). Biochemical characterization of the purified enzyme revealed that this novel protease was relatively oxidant stable and moderately halo- and thermo-tolerant. This is the first report on the biochemical characterization of the recombinant AprX-related serine protease. In addition, the evolutionary relationship of this enzyme with other AprXs from different bacteria and other enzymes in the subtilases superfamily was studied.

## Results

### Genomic DNA library construction and screening

Screening of a *Virgibacillus *sp. SK37 genomic DNA expression library in *E*. coli DH10B was done on LB-skim milk agar plates as described in the materials and methods section. An enumeration analysis determined the size of the genomic library to be 1.56 × 10^5 ^clones with 15% background (blue colonies harboring self-ligated plasmid), yielding 1.33 × 10^5 ^clones with inserts. When an average size of 4.4 × 10^6 ^bp of bacilli genomic DNA was considered (calculated from whole genome sizes available from the NCBI database), this library theoretically covered the genome size of at least 91 and 302 times (depth of coverage) when the DNA fragments of 3,000 and 10,000 bps, respectively, were taken into account. Of approximately 100,000 clones screened (accounting for more than 68 depth of coverage), six clones showed clear halo zones, indicating the presence of proteolytic activity. The recombinant plasmids were individually extracted from these six clones and their DNA inserts were analyzed. Restriction digestion analysis showed that among these 6 clones, there were four different DNA inserts based upon size: ~ 3,300 bp (1 clone), ~3,800 bp (1 clone), ~4,300 bp (3 clones), and ~5,300 bp (1 clone). Remarkably, nucleotide sequence analysis of these clones indicated that they all shared overlapping nucleotide sequences, resulting in an open reading frame (ORF) of only one putative gene.

### Nucleotide and deduced amino acid sequences analysis

**Figure 1:**
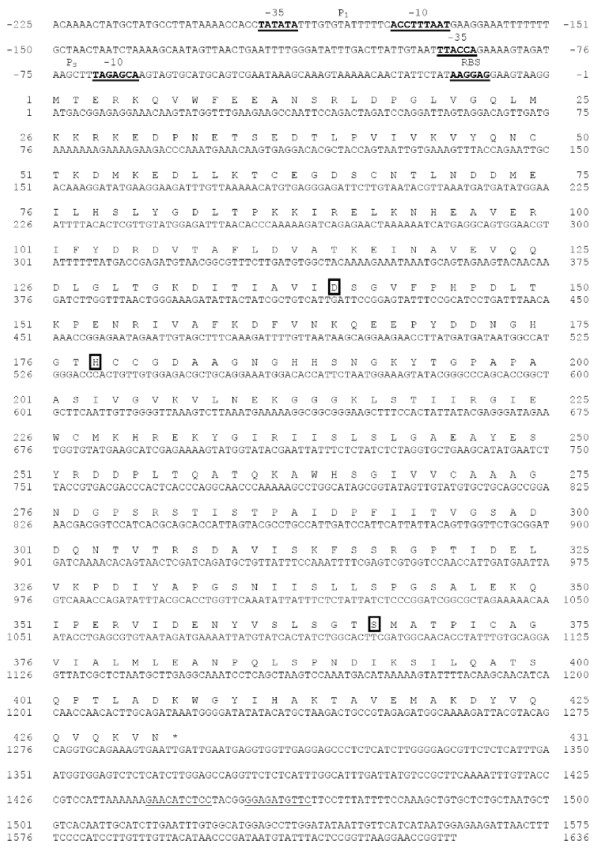
**Nucleotide and deduced amino acid sequences of the gene aprX-sk37**. Translation starts at position 1. The *aprX-sk37 *gene comprises a 1293 bp ORF encoding 431 amino acid residues, indicated as single-letters above the nucleotide sequence. The nucleotide and predicted amino acid are numbered on the right and left sides. The predicted -35 and -10 regions of the putative short and long promoters (P_S _and P_L_) are presented in bold. The putative ribosomal binding site (RBS) and the transcription terminator sequences are underlined. Residues involving in the hypothetical catalytic triad, Asp_140_, His_178_, and Ser_367_, are boxed. The stop codon is indicated by an asterisk.

**Figure 2:**
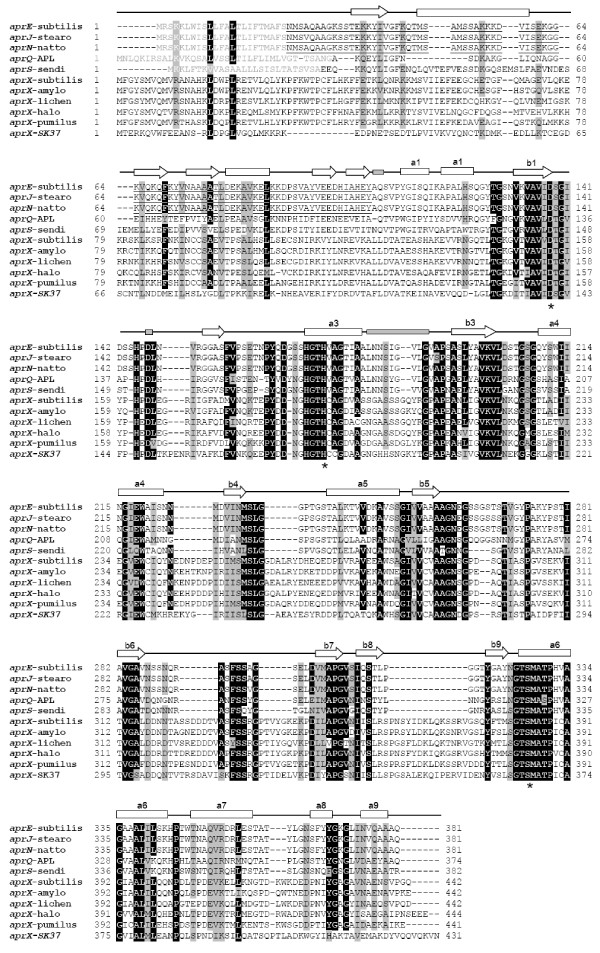
**Multiple amino acid sequence alignment of AprX-SK37 with selected Apr-related subtilisin-like serine proteases**. Sequence aligned: subtilisin E (*aprE*-subtilis) (P04189) from *Bacillus subtilis*; Subtilisin J (*aprJ*-stearo) (P29142) from *B. stearothermophilus*; subtilisin NAT (aprN-natto) (P35835) from *B. subtilis *subsp. "natto"; subtilisin ALP (aprQ-APL) (Q45523) from *Bacillus *sp. GN; subtilisin Sendai (*aprS*-sendi) (Q45522) from *Bacillus *sp. G-825-6; AprX (*aprX*-subtilis) (O31788) from *B. subtilis*; AprX (*aprX*-amylo) (A7Z4Z4) from *B. amyloliquefaciens*; AprX (*aprX*-lichen) (Q65IP4) from *B. licheniformis*; intracellular alkaline serine protease (*aprX*-halo) (Q9KBJ7) from *B. halodurans*; S8A subfamily protease (*aprX*-pumilus) (A8FDI1) from *B. pumilus*; alkaline serine protease X (*aprX-sk37*) from *Virgibacillus *sp. SK37. Secondary structure elements of the AprE are indicated above the alignment; common structural elements are shown as; rectangular for α-helices, arrow for β-sheet strands, and filled thin bar for calcium binding domain [42]. Identical and similar (>75%) amino acid residues among all enzymes are shaded in black and gray, respectively. Conserved amino acid residues involved in the active side (Asp, His, and Ser) are indicated by asterisks below the alignments. Signal and pro-peptide are marked by a gray-letter and underline, respectively.

The nucleotide and deduced amino acid sequences of the isolated ORF and its flanking sequences are shown in Figure 1. The ORF begins with the ATG start codon at nucleotide position 1 and terminates with the TGA termination codon at nucleotide position 1,294. A sequence with good consensus to the canonical ribosomal binding-site (AAGGAG) was found 10 bp upstream from the ATG start codon. Two putative promoters (P_L _and P_S_) showing moderate consensus to the σ^A ^promoter corresponding to the -10 and -35 regions were located at nucleotide positions -166 and -189 for the P_L _and -63 and -81 for the P_S_, respectively (Figure 1). An inverted-repeat sequence from nucleotide 1,440 to 1,465 is found 143 bp downstream of the termination codon. The ORF in the nucleotide sequence encodes 431 amino acids with a calculated molecular mass (MM) of 47,100 Da, and predicted p*I *of 5.2. Comparison of the amino acid sequence of this enzyme with other proteases in the subtilase superfamily is shown in table 1. Except for subtilase family D, the deduced amino acid sequence exhibited resemblance to members of the subtilase superfamily (subtilisin-like proteases) with 20 to 27% identity as determined by ClastalW2. In addition, the coding enzyme shows a limited similarity to the AprXs from *Bacillus *sp. SG-1 (ZP_01859218), *B. licheniformis *ATCC 14580 (YP_079346), and *B. amyloliquefaciens *FZB42 (YP_001421301) with 45, 43, and 42% identity, respectively. Apr-related enzymes are alkaline serine proteases encoded from the *apr *gene found mostly in the class bacilli. To date, a number of *apr *genes have been identified such as *aprA *(Alkaline protease from *B. thuringiensis*), *aprE *(Subtilisin E from *B. subtilis*; AprE from *B. amyloliquefaciens*; M-protease from *B. clausii*; Elastase from *B. cereus*), *aprN *(Subtilisin Nattokinase from *B. subtilis *subsp. "natto"), *aprJ *(Subtilisin J from *B. stearothermophilus*), *aprQ *(Subtilisin ALP from *Bacillus *sp.), *aprM *(AprM from *Bacillus *sp.), *aprS *(Subtilisin Sendi from *Bacillus *sp. G-825-6), and *aprX *(*i.e.*, AprX from *B. subtilis*) [23]. Herein, we designated our recently isolated gene from *Virgibacillus *sp. SK37 as *aprX-sk37 *and the encoded enzyme as AprX-SK37. The DNA sequence of this novel enzyme has been submitted to GenBank and its accession number is HM587897. Multiple amino acid sequence alignment of the Apr-related enzymes obtained from the SwissProt database and AprX-SK37 is shown in Figure 2. The similarity to other AprX enzymes starts from residue 130 of AprX-SK37 and extends nearly to the C-terminus of the protein. Aspartic acid, histidine, and serine in the catalytic triad and asparagine in the oxyanion hole of subtilases are fully conserved in the AprX-SK37 sequence (Asp_140_, His_178_, Ser_367_, and Asn_276_, respectively, according to the AprX-SK37 numbering). All common secondary structure elements of enzymes belonging to the subtilases [4] could be found in the catalytic core of AprX-SK37. When alignment gaps of at least three amino acids are considered, among the AprX sequences, AprX-SK37 shows unique insertion and deletion regions. The deletions are between Lys_29 _and Glu_30 _(12 residues), Gly_235_, and Ile_236 _(3 residues), while the insertion is found from Pro_152 _to Asn_154 _(3 residues) according to AprX-SK37 numbering. AprX-SK37 lacks a canonical signal sequence for membrane translocation (signal peptide) at its N-terminus, indicating an intracellular location as suggested by sub-cellular prediction servers of SignalP 3.0 [24] and ProtCompB (Softberry Bioinformatics tools: http://linux1.softberry.com/berry.phtml). This prediction agreed with all known AprXs, which lack N-terminal signal sequences for secretion as well.

**Table 1:** Homology analysis of AprX-SK37 and other enzymes in the subtilase superfamily

Family	Protein name	Organism	UniProt ID	Identity (%)	Similarity (%)	References
A	Subtilisin E	*Bacillus subtilis *subsp. subtilis 168	P04189	26.9	32.9	[50]
B	Thermitase	*Thermoactinomyces vulgaris*	P04072	23.7	23.7	[51]
C	Proteunase K	*Tritirachium album *limber	P06873	22.3	31.6	[52]
D	lantibiotic leader peptidase	*Lactobacillus sakei *L45	Q48854	9.5	26.7	[53]
E	kexin	*Saccharomyces cerevisiae*	P13134	20.9	44.5	[54]
F	pyrolysin	*Pyrococcus furiosus*	P72186	27.4	38.1	[55]

Note: Percent identity and similarity were calculated by the ClastalW2 and expressed as percentage compared to AprX-SK37 sequence. Protein ID(s) were retrieved from the UniPort databank (http://www.uniprot.org/)

### Phylogenetic tree analysis

**Figure 3:**
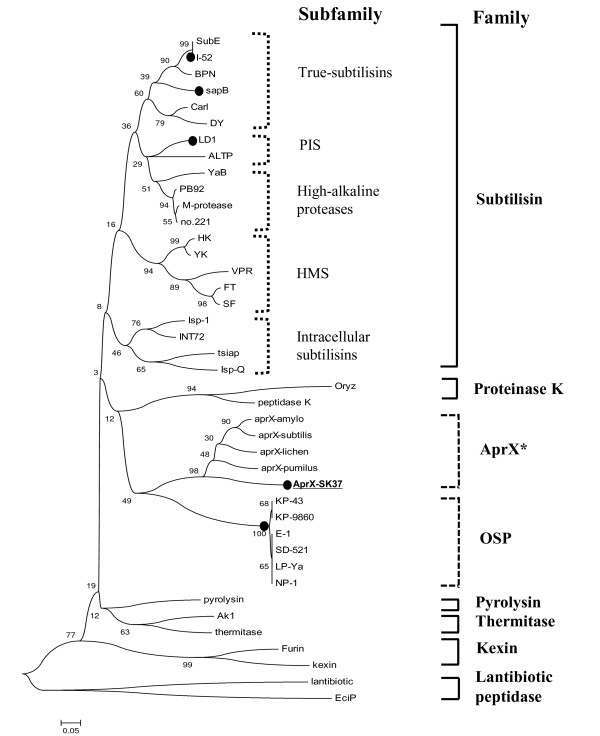
**Phylogenetic tree of enzymes in the subtilase superfamily based on amino acid sequence similarity in catalytic domains**. The bootstrap consensus tree inferred from 1000 replicates was taken to represent the evolutionary history of the sequence analyzed. Branches corresponding to partitions reproduced in less than 50% bootstrap replicates are collapsed. The percentage of replicate trees in which the associated sequence clustered together in the bootstrap test (1000 replicates) is shown next to the branches. All positions containing gaps and missing data were eliminated from the dataset (Complete deletion option). There were a total of 66 positions in the final dataset. The subtilase superfamily is classified as recommended by Siezen et al [4] (square brackets). The subtilisin family is subdivided into true subtilisins, phylogenetically intermediate subtilisins between true subtilisins and high-alkaline protease (PIS), high-alkaline proteases, high-molecular-mass subtilisins (HMS), and intracellular subtilisins [17] (dotted square brackets). AprX-SK37 from *Virgibacillus *sp. SK37 in this study (underlined) is a member of the novel AprX family. Black dots indicate oxidant stable proteases (OSPs). The newly classified alkaline serine protease X (AprX) family (dashed square bracket), proposed in this study is marked with a star.

The predicted amino acid sequence of AprX-SK37 was aligned and compared with those of well-characterized proteases in the subtilase superfamily retrieved from MEROPS [5] and the SwissPort [23] databases. Oxidant stable proteases (OSPs) from various strains were also included in the alignment (indicated by black dots). The phylogenetic tree was inferred using the neighbor-joining algorithm, as shown in Figure 3. The Sequences aligned are: subtilisin E (SubE) (P04189) from *Bacillus subtilis *168; I-52 from *B. clausii *I-52 [25]; BPN' (P00782) from *B. amyloliquefaciens*; sapB (CAO03040) from *B. pumilus*; Carlsberg (Carl) (P00780) from *B. licheniformis*; DY (P00781) from *B. subtilis *DY; ALTP (BAF34115) from *Alkaliphilus transvaalensis*; LD1 (BAD02409) from *Bacillus* sp. strain KSM-LD1; YaB (P20724) from *Bacillus* sp. strain YaB; PB92 (P27693) from *B. alkalophilus *PB92; M-proteinase (Q99405) from *B. clausii *KSM-K16; no. 221 (P41362) from *Bacillus* sp. strain 221; HK (BAD36786) from *Bacillus *sp. strain D6; YK (BAD36788) from *Bacillus *sp. strain Y; VPR (AAA22881) from *B. subtilis*; FT (BAD21124) from *Bacillus *sp. strain KSM-KP43; SF (BAD21125) from *Bacillus *sp. strain KSM-9865; Isp-1 (AAA22557) from *B. subtilis*; INT72 (P29139) from *B. polymyxa *72; tsipa (BAA13418) from *Thermoactinomyces sp*.; Isp-Q (BAA07142) from *Bacillus *sp. strain NKS-21; KP-43 (BAB55674) from *Bacillus *sp. strain KSM-KP43; KP-9860 (BAB21266) from *Bacillus *sp. strain KSM-KP9860; E-1(BAB21265) from *Bacillus *sp. strain D-6; SD-521 (AB046402) from *Bacillus *sp. strain SD-521; LP-Ya (AB046404) from *Bacillus *sp. strain Y; NP-1 (BAB21269) from *Bacillus *sp. strain NV1; Oryzin (Oryz) (P35211) from *Aspergillus flavus*; Peptidase K (P06873) from *Engyodontium album*; pyrolysin (P72186) *Pyrococcus furiosus*; Thermitase (P04072) from *Thermoactinomyces vulgaris*; subtilisin AK1 (AK1) from *Geobacillus stearothermophilus*; kexin (P13134) from *Saccharomyces cervisiae*; furin (P09958) from *Homo sapiens*; LasPlantibiotic (lantibiotic) (Q48854) from *Lactobacillus sakei*; EciPlantibiotic (EciP) (O54221) from *Staphylococcus epidermidis*; and AprX-SK37 (HM587897).

The subtilase superfamily was phylogenetically grouped into six clusters: subtilisins, thermitase, proteinase K, lantibiotic peptidases, kexin, and pyrolysins (indicated by solid square brackets), which represent subtiliase family S8A, B, C, D, E, and F, respectively, [4]. From our analysis, AprX-SK37 was not categorized into any of these families, but shared the same cluster with other AprXs, which was positioned apart from other subtilase families (dashed square bracket). Notably, within the AprX cluster, AprX-SK37 is relatively distant from other AprXs. Most OSPs that are produced from alkaliphilic bacilli are clustered together in the tree (dashed square bracket), *i.e*. E-1, LP-Ya, and SD-521 from *B. cohnii *DSM 6307, NP-1 from *B. halmapalus *DSM8723, KP-43 from *Bacillus* sp. KSM-KP43 [17]. However, some of OSPs such as sapB (AM748727) from *B. pumilus *CBS [15], LD1 (AB085752) from *Bacillus *sp. KSM-LD [8], and I-52 from *B. clausii *I-52 [16,25], were not located in the OSPs cluster, but appeared to be members of the subtilisin family.

### Over-expression and purification of recombinant *Virgibacillus *AprX-SK37

**Figure 4:**
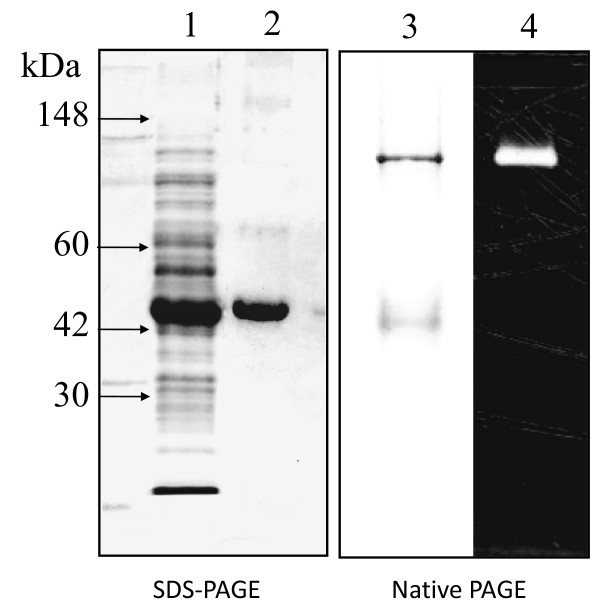
**SDS-PAGE and casein-zymography of recombinant AprX-SK37**. Samples were electrophoresed on a 10-15% SDS polyacrylamide gel (lane 1 & 2) and 12% native polyacrylamide gel (lane 3 & 4). After electrophoresis, the gel was stained with Coomassie brilliant blue (lanes 1 - 3) or detected for protease activity (lane 4). Lane 1, whole cell lysate obtained from *E. coli *BL21 harbouring plasmid pET-AprX-SK37; lane 2, purified recombinant AprX-SK37; lane 3, purified recombinant AprX-SK37 run in a native polyacrylamide gel; lane 4, purified recombinant AprX-SK37 in casein-zymogram. Standard molecular weights for SDS-PAGE (lane 1 & 2) are marked by arrows.

To express the recombinant *Virgibacillus *AprX-SK37, the *aprX-sk37 *gene (nucleotide no. -39 to 1293, according to Figure 1) was amplified by PCR and cloned into the *Xba*I and *Xho*I restriction sites of pET21d(+) vector such that the ribosomal binding site (RBS) and the start codon of the vector were replaced by those of the native gene. Notably, the RBS of the expression vector and *aprX-sk37 *gene are identical (AAGGAG). The recombinant plasmid, pET-AprX-SK37 was expressed using *E. coli *BL21(DE3) as an expression host. *E. coli *BL21/pET-AprX-SK37 was cultured as described in the materials and methods section. To optimize the induction condition, samples from various fractions including inclusion body, cytoplasm, periplasmic space, and culture supernatant taken at various time points after induction with different concentration of IPTG were analyzed by SDS-PAGE and activity assay. *E. coli *BL21 carrying empty pET21d (+) vector was used as a control, of which no significant enzymatic activity could be detected. Upon induction with 0.1 mM IPTG, the recombinant enzyme could only be found in the cytoplasmic fraction as soluble protein. No enzyme could be detected in the periplasmic extract or the culture supernatant (data not shown). Neither lowering the temperature nor varying the concentration of IPTG dramatically affected expression level of the enzyme as determined by SDS-PAGE (data not shown). Routinely, ~16 mg of purified recombinant AprX-SK37 could be obtained from 1-liter culture. The C-terminal 6xHis-tagged enzyme could be purified from cleared cell lysate by one step affinity chromatography using Ni-NTA resin in native condition to apparent homogeneity as shown in Figure 4 (lane 1 and 2). The purified AprX-SK37 showed a MM of 46 kDa on SDS-PAGE, which corresponded well to the theoretical mass of 47 kDa. In the native PAGE analysis, proteolytic activity could be observed by casein-zymogram (Figure 4, lane 4) at the corresponding position on the Coomassie stained gel (Figure 4, lane 3). Zymographic assay in a denatured condition was impracticable due to enzyme sensitivity towards SDS, even though an in-gel refolding step was performed (data not shown).

### Effects of pH and temperature

**Figure 5:**
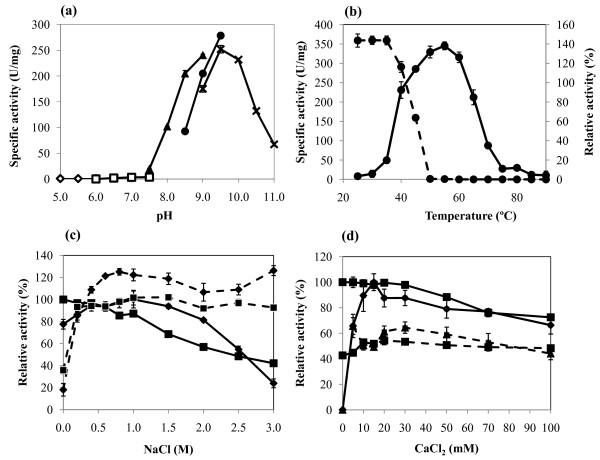
**Effects of pH (a), temperature (b), NaCl (c), and CaCl_2 _(d) on the activity (solid lines) and stability (dashed lines) of recombinant AprX-SK37**. In (a), pH profile was carried out in acetate (white diamond), Tris-maleate (white square), Tris-HCl (black triangle), Tris-glycine (black circle), and glycine (cross) buffer. In (b) the temperature profile and thermostability of AprX-SK37 are illustrated by solid line and dashed line, respectively. In (c) and (d), the effects of sodium and calcium on AprX-SK37 (black diamond) and subtilisin A (black square) activities are shown. Enzyme stability (dashed line) was assayed by pre-incubating the enzymes at different concentrations of NaCl or CaCl_2 _at 25°C for 24 h and expressed as relative activities compared to the reaction at the corresponding concentrations of NaCl or CaCl_2 _without pre-incubation.

The optimal pH of AprX-SK37 was determined based on azocaseinolytic activity. The enzyme is more active at pH 8.5 and 9.0 when using Tris-HCl buffer than Tris-glycine buffer. However, AprX-SK37 showed a maximal activity at pH 9.5 in the Tris-glycine buffer (Figure 5a). No activity was found at pH lower than 7.5, indicating an alkaline protease characteristic. Azocaseinolytic activity was maximum around 55°C (Figure 5b). The enzyme appeared to be activated after pre-incubation for 2 h at 25-30°C prior to the assay. Whereas, after 2 h of pre-incubation time, 50% and 0% residual activity was detected at 47°C and beyond 50°C, respectively.

### Effects of NaCl and CaCl_2_

Proteases produced from *Virgibacillus *sp. SK37 have been shown to exhibit NaCl- and CaCl_2_-activated characteristics [26,27]. Therefore, the effects of NaCl and CaCl_2 _on the activity of AprX-SK37 were studied. As demonstrated in Figure 5c (diamond, solid line) the activity of AprX-SK37 increased slightly when NaCl was added, and showed maximum activity at 1 M. The activity decreased to 30% at 3 M NaCl. On the contrary, subtilisin A, which was used as a control, did not show NaCl-activated characteristics as indicated by a decrease in activity with increasing concentration of NaCl as shown in Figure 5c (square, solid line). Both AprX-SK37 and subtilisin A appeared to require at least 0.25 M of NaCl to retain their original activities after incubation at 25°C for 24 h (Figure 5c, dashed lines). When the AprX-SK37 enzyme was first pre-incubated with 0.5 - 3 M NaCl for 24 h at 25°C and pH 9.5, the activity was enhanced up to 125% (Figure 5c, diamond, dashed lines). These NaCl-enhancing stability effects could not be observed in subtilisin A (Figure 5c, square, dashed lines).

In addition to NaCl, AprX-SK37 also showed a CaCl_2_-dependent characteristic as shown in Figure 5d (diamond, solid line). The enzyme was inactive in the absence of Ca^2+ ^and required 15 mM CaCl_2 _for its maximal activity. This is opposite to the Ca^2+ ^independent charateristic of subtilisin A (Figure 5d, square, solid line). The activities of both AprX-SK37 and subtilisin A gradually decreased when the CaCl_2 _concentration was higher than 30 mM. The remaining activities of AprX-SK37 and subtilisin A, when CaCl_2 _reached 100 mM, were 66 and 73%, respectively. Both enzymes showed moderate stability (50% remaining activity) after pre-incubation for 24 h at 25°C.

### Effects of reducing agents and various inhibitors

The effects of various inhibitors, reducing agents and detergent on AprX-SK37 activity were reported in Table 2. A marked inhibition by 10 mM PMSF suggested that this enzyme is a serine protease. However, no inhibition by other serine protease inhibitors, *i.e*. TLCK, TPCK, leupeptin, and SBTI could be observed. A strong inhibition by EDTA indicated a metal-dependent character of this enzyme. However, bestatin, a metalloprotease inhibitor selective for aminopeptidases, had no inhibitory effect. The enzyme was very sensitive to detergent (SDS), and reducing agents such as DTT and BME, whereas the inhibitor E-64, which is an active-site titrant of cysteine proteases, as well as IAA and NEM, did not affect enzyme activity (Table 2). Note that CaCl_2 _was routinely added to the standard enzyme assay.

**Table 2:** Effects of protease inhibitors and denaturants on recombinant AprX-SK37 activity using azocasein as a substrate^a^

Characteristic	Inhibitors	Concentration	Remaining activity (%)
Serine protease inhibitor	Leupeptin	10 mM	100.7
	SBTI	0.4 mg/ml	100.9
	TLCK	10 mM	99.4
	TPCK	10 mM	99.9
	PMSF	1 mM	89.2
		10 mM	1.0

Metalloprotease inhibitor	EDTA	0.5 mM	78.1
		1 mM	64.2
		5 mM	1.4
	Imidazole	0.5 M	82.4
	L-Histidine	0.5 M	108.1

Cysteine protease inhibitor	IAA	0.1 M	98.0
	NEM	0.1 M	114.2
	E64	1 mM	99.2
	
Aspartic protease inhibitor	Pepstatin A	1 mM	102.1

Amino peptidase inhibitor	Bestatin	2 mM	102.6

			
			

Denaturants	Substances	Concentration	Remaining activity (%)

H-bond breaker	Urea	1M	20.8
		2M	0.0

Detergent	SDS	0.5%	0.0
		1.0%	0.0

Reducing agents	DTT	1 mM	38.5
		10 mM	2.3
		20 mM	0.1
	BME	1 mM	64.7
		10 mM	41.9
		20 mM	14.1

^*a *^The enzymatic activity was determined at pH 9.5 and 55°C in the presence of 5 mg/ml substrate and 5 mM CaCl_2_. The experiment was performed in triplicate, and standard errors from the means were within 15% of the reported values.

### Thermal stability

**Figure 6:**
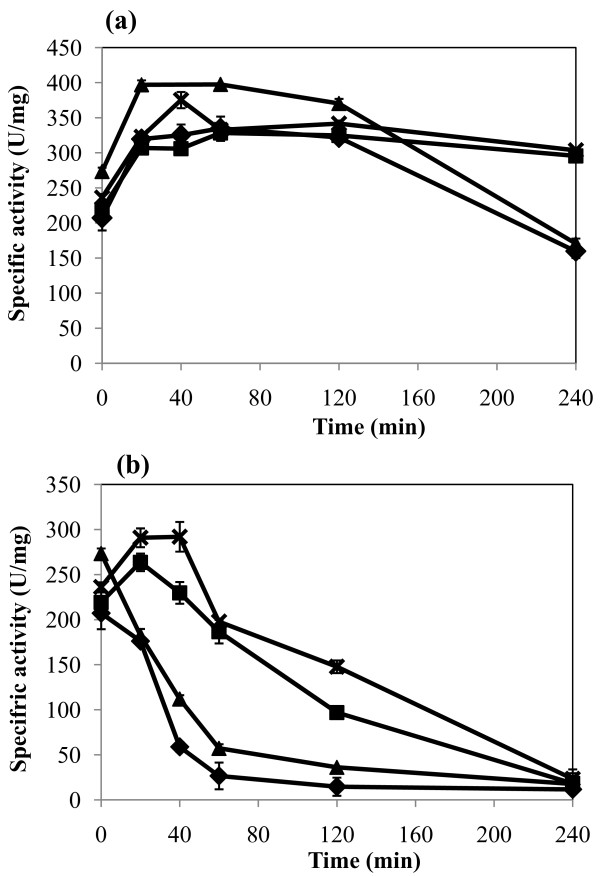
**Effects of NaCl and CaCl_2 _on thermostability of recombinant AprX-SK37**. The enzyme was pre-incubated at either 40 (a) or 55°C (b) in 100 mM Tris-Glycine pH 9.5 containing; 5 mM CaCl_2 _(black triangle): 5 mM CaCl_2 _and 1 M NaCl (black square): 15 mM CaCl_2 _(black diamond): 15 mM CaCl_2 _and 1 M NaCl (cross). Residual enzyme activities were measured at 55°C under standard assay conditions.

Previous report has suggested that mono and divalent cations contribute to the stability of the native state and increase the activation energy of unfolding of subtilisin BPN [28], therefore we decided to investigate the thermostability of AprX-SK37 in the presence of various concentrations of Ca^2+ ^and Na^+ ^cations. At 40°C, the enzyme activity could be activated by calcium. In the standard reaction condition (5 mM CaCl_2_) the enzyme was activated after incubation for 30 min and the optimal activity was retained for up to 2 h (Figure 6a, diamond). When the concentration of CaCl_2 _was increased to 15 mM (triangle), the highest enzyme activation could be observed from 0.5-2 h. When the enzyme was incubated longer than 2 h in both 5 and 15 mM CaCl_2_, the activity declined. Moreover, when 1 M NaCl was added in the presence of either 5 mM (square) or 15 mM (cross) CaCl_2_, the stability of the enzyme was increased up to 4 h. (Figure 6a). The same phenomenon but at a higher rate of thermal inactivation was observed at 55°C (Figure 6b). The enzyme activity in the presence of only CaCl_2 _(either 5 mM or 15 mM) decreased rapidly after the first 1 h of incubation and became almost completely inactivated after 2 h (Figure 6b, diamond and triangle). In contrast, addition of 1 M NaCl could significantly lower the rate of thermal inactivation as compared to those without NaCl. The residual activity of the reaction containing 1 M NaCl + 5 mM or 15 mM CaCl_2 _at 2 h was approximately 45% and 60%, of their original activity, respectively (Figure 6b, cross and square). These observations suggested that NaCl played an important role in exerting enzyme thermal stability while CaCl_2 _was only responsible for activating enzyme activity.

### Effect of oxidizing agent

**Figure 7:**
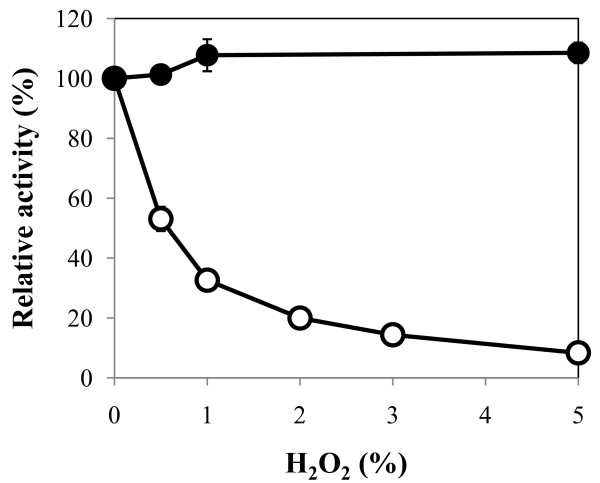
**Stability of recombinant AprX-SK37 towards H_2_O_2_**. The activity was determined at various H_2_O_2 _concentrations after pre-incubation for 0 (black circle) and 30 (white circle) min. The activity without H_2_O_2 _was taken as 100%.

Stability against oxidizing agents is one of the most attractive properties of bacterial proteases. Therefore, we used H_2_O_2 _as the representative oxidizing agent to test AprX-SK37 oxidant stability. As shown in Figure 7, the enzyme was fully active in the presence of as much as 5% H_2_O_2_. However, the enzyme activity gradually decreased when it was pre-incubated for 30 min with increasing concentration of H_2_O_2_. No activity was found in a reaction pre-incubated with 10% H_2_O_2 _(data not shown)

## Discussion

This research reports the cloning, expression, biochemical characterization, and phylogenetic analysis of a novel intracellular bacterial AprX serine protease. A previous report [29] of this enzyme from *B. subtilis*, focusing on bioinformatic analysis and regulation of gene expression, indicated that this enzyme is an intracellular serine protease, which is expressed in the stationary phase and not essential for either growth or sporulation. The AprX-SK37 enzyme in this study was derived from a moderately halophilic bacterium, *Virgibacillus *sp. SK37, which was isolated from Thai fish sauce that was saturated with 26-28% of NaCl. This bacterium produced numerous NaCl-activated subtilisin-like proteinases, three of them having been partially purified and characterized as extracellular halotolerant bacillopeptidase F-like proteinases [30]. Their activities did not depend on calcium, showed maximum activity at pH 8, 55-60°C, 25-30% NaCl and 70-100 mMol l^-1 ^CaCl_2_, and stability at 0-30% NaCl and <20 mMol l^-1 ^CaCl_2 _[30]. The NaCl-activated property of AprX-SK37 was not unexpected as it has been shown that the cell membrane of halophilic bacteria is incapable to completely prevent an increased flux of sodium into the cells [31]. Intracellular Na^+ ^concentrations of moderately halophilic bacteria growing in media containing 2-4 M NaCl were found between 0.13 to 3.52 M with an average of 1.37 M [32]. Our enzyme can be activated and stabilized by 1 M NaCl (5.8% w/v) and tolerate up to 3 M NaCl (17.4% w/v). To the best of our knowledge, this is the first report on a halotolerant intracellular serine protease.

DNA sequence analysis of *aprX-sk37 *indicated that the gene is regulated by two putative σ^A ^promoters (P_L _and P_S_), which have been suggested to function as primary and secondary promoters of *B. subtilis aprX*, respectively [29]. The open reading frame (ORF) of *Virgibacillus aprX-sk37 *is slightly smaller than that of *B. subtilis aprX *and lacks the alternative start condon TTG, at position 76-78, Met/Leu_26 _in AprX numbering of *B. subtilis*. The predicted MM and p*I *of AprX-SK37 are in the same range as those of AprXs from *Bacillus *species (MM of 42.2-48.9 kDa and p*I *at 4.9-6.1). The key difference between AprX-SK37, other AprXs, and some other intracellular proteases is its N-terminal domain, which showed no similarity to those of known proteins in the database. It has been suggested that the N-terminal domain of subtilases acts as an intramolecular chaperone or pro-peptide that mediates enzyme folding; nevertheless, the role of this domain on AprX-SK37 activity is unclear. The localization of AprX from *B. subtilis *in cytoplasm has been previously reported [33]. The recombinant AprX-SK37 in this study is found in cytoplasm; however, the precise localization of the wild-type enzyme is unknown. The enzyme appeared to be active without cleavage of any pro-peptide. Its putative function as a chaperone for correct enzyme folding or as a regulatory domain remains to be explored.

The benefit of isolating a novel enzyme from a DNA expression library instead of PCR-cloning using degenerated primers is that the gene encoding active enzyme can be readily obtained. This advantage is very important especially for *Bacillus *subtilases, which often form inactive, insoluble aggregates when produced in *E. coli *expression systems [34-36]. The recombinant AprX-SK37 appeared to be located as active soluble protein in the cytosolic compartment; none were found as inactive, insoluble inclusion bodies or secreted into the periplasm or culture media. The fact that the enzyme was first identified from *E. coli *colonies that showed halo zone during genomic DNA library screening could be the result of membrane leakage after prolonged growth for more than 24 h. Remarkably, despite a thorough screening of the library that covered approx. 70 depth of the library, no other protease genes could be isolated. This could be because other enzymes formed inactive aggregates, are unstable in *E. coli*, or the enzyme activity may require higher sodium or calcium, which was not included in the screening condition. Moreover, it could be because the active enzymes might need specific post-translational modification, which was lacking in the *E. coli *expression system and that the substrate in our screening (milk protein) was not an appropriate substrate for certain enzymes. Further modification of screening conditions may lead to the identification of additional active enzymes. pET vector was used to express the recombinant *aprX-sk37 *gene in this study. This system has been shown to be efficient for the expression of various *Bacillus *hydrolytic enzymes [37,38]. The canonical ribosomal binding site in this vector is identical to that of *Virgibacillus aprX-sk37*. The high level of expression could be because of synonymous codon usage, which leads to a fast translation rate in the *E. coli *expression system [39].

Phylogenetic analysis revealed that AprX-SK37 belongs to the superfamily of subtilases or subtilisin-like proteases. This result is consistent with the family analysis of *B. subtilis *AprX [29]. Moreover, our analysis indicated that the AprXs are clustered in a distinct group, of which AprX-SK37 is relatively different from. This could be because AprX-SK37 possesses unique characteristics of oxidant stability and moderate salt-activation, which are not found in other AprXs. However, according to our phylogenetic tree, AprX-SK37 was not categorized into the OSPs group. Notably, phylogenetic analysis of OSPs reports little evolutionary relationship among this group of enzymes. Our phylogenetic analysis indicated that most OSPs do not belong to any of the subtilisin families previously described in bacteria or fungi, but appear to belong to a different cluster (designated OSP in Figure 3). This finding is similar to the phylogenetic analysis of a subtilisin-like protease gene family in the grass endophytic fungus *Epichloë festucae *[40]. Hence, OSPs should not be classified as the subfamily of subtilisins as previously suggested [11,12]. Phylogenetic analysis of various OSPs producers suggested that the sources of OSPs encompass bacilli from diverse taxon, which could be the result of different mechanisms of oxidant stabilization. Taken together the result of phylogenetic analysis as discussed above, we would like to propose a new family of subtilases superfamily, namely alkaline serine protease X (AprX) or subitlase S8G, according to the MEROPS database derived from subtilases classification by Siezen and Leunissen [4]. In addition, we believe that the classification of OSPs has to be re-defined.

Biochemical characterization revealed that recombinant AprX-SK37 is a Ca^2+^- dependent alkaline serine protease and moderate thermophile with optimal pH and temperature at 9.5 and 55°C, respectively. The enzyme requires calcium to function with maximum activity at 15 mM CaCl_2_. A Calcium-dependent characteristic is well known for members of the subtilases superfamily, of which conserved calcium binding sites are essential for enzyme stability and/or activity [4]. In nature, calcium may play a physiological role in *aprX-sk37 *gene regulation as it has been shown that a massive accumulation of calcium during sporulation is found in most bacilli in order to respond to adverse environmental changes [41]. In *B. subtilis*, AprX is expressed in the late stationary phase under the control of various transition state regulatory genes [29]. The high sensitivity of AprX-SK37 to a reducing agent suggested that disulfide bonds are essential for structural stability. The primary amino acid sequence revealed that AprX-SK37 contains 8 cysteines (Cys). This richness in Cys is unique for enzymes in the AprX family since Cys is usually absent from subtilases in Gram-positive bacteria [4,29].

In general, subtilases are inactivated by hydrogen peroxide due to the oxidation of methionine (Met), located next to the catalytic serine (Met_222 _in subtilisin BPN numbering), thus preventing the formation of the tetrahedral intermediate during the transition state of their hydrolytic reaction [42,43]. AprX-SK37 possesses Met_368 _next to catalytic Ser_367_, similar to other subtiliases (Figure 2) and other OSPs [8,11,12,14-16]. The actual mechanism of oxidant stability is still unclear. In the study of KP-43, an OSP enzyme, oxidation of Met was not a fatal modification, but rather altered its substrate specificity [44]. KP-43's crystal structure suggested that the rate of Met-oxidation in KP-43 was lower than those of other subtilases, probably due to the longer distance between the Met residue in the catalytic vicinity and the oxyanion hole [17]. Hence, the oxidant-stable ability is strongly dependent on enzyme conformation and structural integrity, which is intrinsic to each enzyme. The oxidant stability as well as halotolerant and moderately thermophilic properties of AprX-SK37 should make this novel enzyme attractive for biotechnological applications in the future.

## Conclusions

A novel oxidant stable, halotolerant and moderately thermophilic alkaline serine protease, named AprX-SK37, has been identified and characterized. Its unique property is attractive for various biotechnological applications.

## Methods

### Materials

Restriction endonucleases, T4 DNA ligase, and calf intestinal phosphatase (CIP) were purchased from New England Biolabs (New England Biolabs, Ipswich, MA). *Pfu *polymerase was obtained from Promega (Promega, Madison, WI). Skim milk was purchased from Merk (MerkKGoA, Darmstadt, Germany). Azocasein and subtilisin A (subtilisin Carlsberg) were purchased from Sigma (Sigma-Aldrich, St. Louis, MO). All other reagents for molecular and biochemical analysis were of molecular and analytical grade, respectively.

### Bacterial strains, plasmids, and growth conditions

*Virgibacillus *sp. SK37 (GenBank/NCBI no. DQ910840) was cultured at 35°C in a modified halophilic medium (1% yeast extract, 0.3% trisodium citrate, 0.2% potassium chloride, 2.5% magnesium sulfate, and 5% NaCl). The pH was adjusted to 7.0 by NaOH. *Escherichia coli *strain DH5α and DH10B (Mega DH10B™ T1^R^electrocomp™, Invitrogen, CA) were used as host strains for cloning and genomic DNA expression, respectively. *E. coli *BL21(DE3) (Novagen, Madison, WI) was used for enzyme expression. They were grown aerobically at 37°C in Luria-Bertani (LB) medium. Plasmid vector pUC19 (New England Biolabs, Ipswich, MA) and pET21d+ (Novagen, Madison, WI) were used for genomic library construction and over-expression, respectively.

### General molecular biology techniques

Cloning, DNA manipulation, and agarose gel electrophoresis were done as previously described [45]. Genomic DNA was isolated from *Virgibacillus *sp. SK37 using a Wizard^® ^genomic extraction kit (Promega, Madison, WI). Plasmid was isolated with Qiagen plasmid preparation kit (Qiagen, Hilden, Germany). PCR products were separated by gel electrophoresis, and bands of the expected size were isolated from the gel using Qiagen QIAquick gel extraction kit (Qiagen, Hilden, Germany). Automated DNA sequencing was carried out by Macrogen (Seoul, Korea), using the primer-walking technique with pUC/M13 forward/reverse primers for pUC19, and T7/SP6 promoter primers for pET21d+ as initial primers, respectively. Enzymes and reagents were used according to the manufacturer's instructions unless otherwise stated.

### Genomic DNA library construction

The genomic DNA of *Virgibacillus *sp. SK37 was partially digested by incubating with 10 units of *Bfu*CI per 150 μg of genomic DNA at 37°C for 20 min. Fragments ranging in size from 3 to 10 kilobase pairs (kb) were isolated from the agarose gel. The plasmid pUC19 was digested with *Bam*HI and 5'-phosphate groups were removed with calf intestinal phosphatase (CIP, New England Biolabs) prior to gel purification using Qiagen QIAquick gel extraction kit (Qiagen, Hilden, Germany). The genomic *Bfu*CI fragments were ligated with dephosphorylated linearized pUC19 by T4 DNA ligase at a ratio of 3:1. This ligation mixture was precipitated by isopropanol and re-dissolved in 30 μl nuclease-free water prior to transform into competent *E. coli *DH10B by electroporation (2 mm cuvette, 2500 V, Electroporator 2510, Eppendorf AG, Hamburg, Germany). The transformants were then spread on LB_Amp _plates containing 2% (w/v) glucose (LB_Amp+Glu_). The plates were incubated for approx. 14 h at 37°C. Colonies growing on the LB_Amp+Glu _plates were scraped from the agar surface and transferred into LB broth containing 15% glycerol and kept at -70°C until use. A small aliquot of the transformants were spread onto LB_Amp _plates supplemented with 1 mM isopropyl-β-D-thiogalactopyranoside (IPTG) and 0.2% (w/v) bromo-chloro-indolyl-galactopyranoside (X-gal) (LB_Amp+IPTG+X-gal_) for enumeration and determination of percentage of blue white colonies, representing DNA library size and % background.

### Screening of protease-producing clones

The clones from genomic DNA library were spread onto LB_Amp _plates supplemented with 3% (w/v) skim milk powder. The clones were grown at 37°C for 18 h. Positive colonies showing protease activity as indicated by the formation of transparent halo zones around the colonies were selected as positive clones after continual incubation of the plates at 30°C for additional 7 h.

### DNA sequence analysis

Plasmid from each protease-positive clone was isolated by Qiagen plasmid preparation kit. Both strands of DNA insert were sequenced by automated DNA sequencing (Macrogen, Korea). The full length DNA insert of each positive-clone, open reading frames (ORFs), and homology of putative coding sequences were analyzed using tools provided by the National Center for Biotechnology Information (NCBI, http://www.ncbi.nlm.nih.gov). Bacterial promoter and transcription termination site of protease coding gene were predicted by Softberry Bioinformatics tools (http://linux1.softberry.com/berry.phtml). Isoelectric point (p*I*) and molecular mass of deduced amino acid sequence were calculated using software from ExPASy Proteomics server (http://ca.expasy.org/tools/pi_tool.html).

### Cloning, over-expression, and purification of the recombinant AprX-SK37

Putative protease gene was amplified by PCR using forward (5'-CTG TGC TCT AGA GCA AAG TAA AAA CAA CTA TTC TAT AAG GAG GAA G-3') and reverse (5'-CTG TGC CTC GAG ATT CAC TTT CTG CAC CTG CTG TAC G-3') primers (the *Xba*I and *Xho*I sites are underlined). Thirty-five cycles of PCR were performed in a thermal cycler (MJ Research PCT-200 Thermal Cycler DNA Engine, GMI Inc, Ramsey, MI) using *Pfu *DNA polymerase. The amplified 1356 bp DNA product flanked by *Xba*I and *Xho*I restriction sites was cloned into the corresponding restriction sites of pET21d (+) vector for the expression of C-terminally 6xHis tagged enzyme. The integrity of the construct, designated pET-AprX-SK37, was confirmed by agarose gel electrophoresis followed by automated double-strand DNA sequencing. To express the enzyme, the pET-AprX-SK37 vector was transformed into *E. coli *BL21 (DE3). A single colony of *E. coli *BL21 harboring pET-AprX-SK37 (BL21/pET-AprX-SK37) was grown at 37°C in LB_Amp _until the OD_600 _reached 0.6-0.7, then 0.1 mM IPTG was added to induce the gene expression. After further incubation at 16°C for 18 h, the cells were harvested and resuspended in lysis buffer (30 mM Tris-HCl, pH 8.0, 0.3 M NaCl, and 10 mM imidazole). Lysozyme was added to a final concentration of 1 mg/ml and incubated on ice for 30 min. Lysozyme-treated cells were broken by sonication on ice for 2 min with a 10 s bursts at 300 W and a 10 s cooling period between each burst. The lysate was clarified by centrifugation at 10,000 × g for 30 min at 4°C. The recombinant 6xHis-tagged enzyme in the cleared lysate was purified using nickel-nitrilotriacetic acid (Ni-NTA) metal affinity chromatography (Qiagen, Hilden, Germany), according to the manufacturer's protocol with some modifications. After the cleared lysate was incubated with Ni-NTA at 4°C for 4 h with rotary shaking, the slurry was loaded into a column and washed with 10 bed volumes of lysis buffer followed by 10 bed volumes of wash buffers (30 mM Tris-HCl, pH 8.0, 1.0 M NaCl) containing 50 and 80 mM imidazole, respectively. The recombinant enzyme was recovered with an elution buffer containing 250 mM imidazole. Protein concentration and purity of the purified protein were analyzed by Bradford method using bovine serum albumin (BSA) as the standard [46], and SDS-PAGE stained with Coomassie Brilliant blue, respectively.

### Amino acid sequence alignment and construction of the phylogenetic tree

Amino acid sequences of proteases similar to the enzyme in this study were retrieved by BLAST search (http://blast.ncbi.nlm.nih.gov/Blast.cgi). Multiple amino acid sequence alignment was done by ClustalX program [47] using sequences of AprX-related enzymes retrieved from SwissProt database (http://www.isb-sib.ch/services/databases). Sequence identity was calculated by ClustalW2. A phylogenetic tree was constructed from sequences of enzymes representing each member of the subtilase superfamily [4] and oxidant stable proteases (OSPs), obtained from extensive search of the Pubmed (http://www.ncbi.nlm.nih.gov/pubmed/) and Swiss-Prot (http://www.isb-sib.ch/services/databases) databases. The phylogenetic tree was constructed based on amino acid similarity within catalytic domains as described by Saeki et al [12], using *MEGA *version 4 software [48] and was inferred by the bootstrapping neighbor-joining method [49], of which sites involving gaps were excluded from the analysis.

### Genbank accession number

The accession number of our recently isolated gene from the *Virgibacillus *sp. SK37 is HM587897 and the protein identification number is ADM26217

### Determination of protease activity

Protease activity was determined using azocasein as a substrate. The standard assay reaction in 500 μl contained 5 mg/ml azocasein, 100 mM Tris-glycine buffer, pH 9.5, 5 mM CaCl_2_, and 20 μg/ml of purified recombinant protease. The mixture was incubated at 55°C for 30 min with vigorous shaking. The reaction was stopped by adding 150 μl of 20% trichloroacetic acid (TCA) and kept on ice for 30 min, followed by centrifugation at 12,500 × g for 30 min. Then, the supernatant (250 μl) was mixed with equal volume of 2.5 M NaOH and the absorbance at 405 nm was measured. Specific activity (U/mg) was defined as an increase of 0.01 OD_405 _units per minute per milligram of enzyme. Protease activity was also analyzed by native-PAGE casein-zymography. Purified protease in a loading buffer without SDS and β-mercaptoethanol was electrophoresed through a 12% polyacrylamide gel under native condition. Subsequently, the gel was submerged in 2% (w/v) casein solution for 30 min at 4°C with gentle shaking. The gel was then transferred into a reaction buffer containing 50 mM Tris-glycine, pH 9.5, 250 mM NaCl, and 5 mM CaCl_2 _and further incubated at 55°C for 90 min, followed by staining with Coomassie blue. The protease activity was determined as a clear band against a blue background.

### Effects of pH and temperature on protease activity

The effect of pH on proteolytic activity was examined in the presence of various 100 mM buffers, namely sodium acetate (pH 5.0-6.0), Tris-Maleate (pH 6.0-7.5), Tris-HCl (pH 7.5-9.0), Tris-glycine (pH 8.5-9.5), and glycine (pH 9.0-11.0), at 55°C and 5 mM CaCl_2_. To study the effect of temperature, the standard assay was applied using Tris-glycine buffer, pH 9.5 at 25-90°C. Thermostability of the protease was analyzed by incubating the enzyme at various temperatures (25-90°C) for 120 min and residual activity was measured at the standard assay condition (55°C, pH 9.5 and 5 mM CaCl_2_) and calculated as a percentage of activity of the enzyme prior to incubation.

### Effects of NaCl and CaCl_2 _on protease activity

Protease activity at various concentrations of either NaCl (0 to 4.0 M) or CaCl_2 _(0 to 100 mM) was measured at 55°C and pH 9.5. Subtilisin A was used as a representative of the subtilisin S8A family, and its catalytic activity was determined at its optimum condition at 60°C, in 100 mM Tris-HCl, pH 8.0 in appropriate concentrations of NaCl and CaCl_2_. The CaCl_2 _concentration was fixed at 5 mM in all NaCl assays. Maximum activity of each protease was defined as 100%. In enzyme stability studies, the enzymes were pre-incubated in the presence of various concentrations of either NaCl (0 to 4 M) or CaCl_2 _(0 to 100 mM) at 25°C for 24 h at pH 9.5 or 8.0 for the purified recombinant AprX-SK37 and subtilisin A, respectively. Residual activities were measured at 55 and 60°C for AprX-SK37 and subtilisin A, respectively, and expressed as relative activity compared to the reaction containing appropriate concentrations of NaCl or CaCl_2 _without pre-incubation.

### Effects of NaCl and CaCl_2 _on thermostability

The effects of NaCl and CaCl_2 _on the thermostability of the recombinant AprX-SK37 was determined by monitoring enzyme activity at moderate (40°C) and high (55°C) inactivation temperatures according to the results from the thermal stability assays. The enzyme was prepared in 100 mM Tris-glycine pH 9.5 containing; (I) 5 mM CaCl_2_: (II) 5 mM CaCl_2 _and 1 M NaCl: (III) 15 mM CaCl_2_: (IV) 15 mM CaCl_2 _and 1 M NaCl. The reactions were pre-incubated at either 40 or 55°C for up to 4 h and aliquots were withdrawn at various time intervals to measure residual activities at the standard assay condition.

### Effects of protease inhibitors and denaturants

The effects of various protease inhibitors and protein denaturants on protease activity were determined using various substances, namely leupeptin, soy-bean trypsin inhibitor (SBTI), L-1-chloro-3-[-4-tosylamido]-7-amino-2-heptanone (TLCK), L-1-chloro-3-[-4-tosylamida]-4-phenyl-2-butane (TPCK), phenylmethanesulfonyl fluoride (PMSF), ethylenediaminetetraacetic acid (EDTA), imidazole, L-histidine, bestatin, pepstatin A, iodoacetic acid (IAA), N-methylmaleimide (NEM), β-mercaptoethanol (BME), dithiothreitol (DTT), E64, urea, and sodium dodecyl sulfate (SDS). Various concentrations of substances used in this study were listed in Table 2. The enzyme was mixed with each substance at room temperature for 30 min before azocasein was added to initiate catalytic reaction under the standard assay condition. Relative activity was calculated using the activity of the enzyme in the absence of these substances as 100%.

### Stability towards oxidizing agent

Hydrogen peroxide (H_2_O_2_) was used as a representative of the oxidizing agent. Protease activity was measured in the presence of H_2_O_2 _at concentrations ranging from 0 to 5% (w/v). Enzyme activity was performed before and after pre-incubation the reaction in the absence of substrate for 30 min at 25°C. The reaction in the absence of H_2_O_2 _and without pre-incubation was taken as 100%.

## Authors' contributions

EP carried out all experimental work and drafted the manuscript. JY supervised and edited the manuscript dealing with enzyme analysis. SR supervised and helped to edit the manuscript dealing with microbiology. MY conceived of the study, and participated in its design and coordination, supervised all molecular biology work and helped to draft and edit the manuscript. All authors read and approved the final manuscript.
